# High Progesterone Receptor Expression in Prostate Cancer Is Associated with Clinical Failure

**DOI:** 10.1371/journal.pone.0116691

**Published:** 2015-02-27

**Authors:** Thea Grindstad, Sigve Andersen, Samer Al-Saad, Tom Donnem, Yury Kiselev, Christian Nordahl Melbø-Jørgensen, Kaja Skjefstad, Lill-Tove Busund, Roy M. Bremnes, Elin Richardsen

**Affiliations:** 1 Dept. of Medical Biology, UiT - The Arctic University of Norway, Tromso, Norway; 2 Dept. of Clinical Medicine, UiT - The Arctic University of Norway, Tromso, Norway; 3 Dept. of Oncology, University Hospital of North Norway, Tromso, Norway; 4 Dept. of Clinical Pathology, University Hospital of North Norway, Tromso, Norway; 5 Dept. of Pharmacy, UiT—The Arctic University of Norway, Tromso, Norway; Innsbruck Medical University, AUSTRIA

## Abstract

**Background:**

Prostate cancer is a highly heterogeneous disease and one of the leading causes of mortality in developed countries. Specific prognostic and predictive markers for prostate cancer patients are still lacking. A causal relationship between androgens and the development of prostate cancer is generally considered biologically plausible, but androgens are not the sole effector in the complexity of prostate carcinogenesis. The aim of this study was to evaluate the prognostic significance of progesterone receptor in tumor tissue of T1-3N0 prostate cancer patients undergoing prostatectomy.

**Methods:**

Tissue microarrays from 535 patients with prostate cancer were constructed. Duplicate cores of tumor cells and tumor stromal tissue from each resected specimen were extracted. Immunohistochemistry was used to evaluate the in-situ expression of progesterone receptor.

**Results:**

In univariate analyses, high tumor cell density (p = 0.006) and high tumor stromal cell density level (p = 0.045) of progesterone receptor were both significantly associated with tumor progression and clinical failure. In multivariate analysis, progesterone receptor expression in tumor cells was an independent negative prognostic factor for clinical failure (HR: 2.5, 95% CI: 1.2–5.2, p = 0.012).

**Conclusion:**

High progesterone receptor density in tumor cells of the prostate cancer tumor is an independent negative prognostic factor for clinical failure.

## Introduction

Prostate cancer (PCa) is one of the leading causes of death amongst men in the western world [1]. The majority of PCa occurs as an indolent form that is unlikely to invade beyond the local tissue environment. A subgroup of PCas, however, displays aggressiveness and metastatic properties. Such cancers result in a rapid disease progression and reduced disease specific survival [2]. Consequently, the clinical course of PCa is highly individual and difficult to predict from the start.

In lack of specific molecular markers as diagnostic and prognostic tools, the detection of PCa and its treatment strategy is still mainly based on the prostate-specific antigen (PSA) value, Gleason score of tumor biopsies and primary tumor (pT)-staging [3]. PSA cannot separate between the different PCa progression patterns. Accordingly, many of the detected PCa cases represent clinically indolent tumors which untreated will remain stable for years [2]. Hence, PSA screening constitutes a risk for overdiagnosis and overtreatment which is associated with a negative impact on quality of life and extensive financial costs [4,5]. The identification of new, improved prognostic and diagnostic biomarkers for PCa is therefore greatly needed.

Sex steroid hormones, such as androgens, estrogens and progesterone, are potent effectors involved in proliferation, differentiation as well as cellular development, and known contributors to the development of different cancers [6]. The metabolic functions of the prostate is under the regulatory control of such sex steroid hormones [7]. A causal relationship between androgens and the development of PCa is, in general, considered biologically plausible [8]. This indicates a crucial role for the androgen receptor in the prostate carcinogenesis and endocrine treatment failure. However, there is mounting evidence that the androgen receptor is not the only effective endocrine receptor in this complex process. Research suggesting the involvement of both the glucocorticoid-, estrogen- and progesterone receptors in this process have been published [9–13].

Progesterone is a 21-carbon hormone synthesized from steroid precursors in various parts of the body, including the testes, adrenal gland, placenta and the glia cells of the brain, in addition to the ovaries [14]. The progesterone receptor (PGR) exists in two isoforms, PGR-A and PGR-B, and both are transcribed from the same gene. It belongs to the same receptor family as the androgen- and oestrogen receptors, which are expressed in both stromal and tumor cells of the PCa tissue [11,13,15–18]. Currently, there is a general agreement of PGR presence in the stromal cells of PCa [10,17,19–23]. Results regarding PGR’s presence in tumor cells, however, are conflicting [9,10,17,19–25]. Thus, the importance of PGR in the human prostate and in prostate carcinogenesis has never been adequately explained. As a consequence we sought to evaluate the expression of PGR in both tumor cells derived from epithelia (TE) and tumor stromal cells (TS) in malignant prostatectomy specimens and found the PGR density level in both TE and TS to be associated with PCa progression.

## Materials and Methods

### Patients, clinical- and histopathological data

671 patients who underwent radical prostatectomies as initial treatment for adenocarcinoma from 1995 to 2005 were retrospectively identified from the Departments of Pathology at the University Hospital of Northern Norway (n = 267), the Nordland Hospital (n = 63) and the St. Olavs Hospital (n = 341). Of these, a total of 136 patients were excluded, due to (i) radiotherapy to the pelvic region prior to surgery (n = 1), (ii) other malignancies within 5 years prior to the PCa diagnosis (n = 4), (iii) inadequate paraffin-embedded tissue blocks (n = 130), and (iiii) lack of clinical follow-up data (n = 1). None of the patients had received hormonal therapy prior to or at the time of the prostatectomy. Thus 535 patients with complete follow-up data were included in this study. Median follow-up time was 89 (range 6–188) months at the last patient update in November 2012. Complete demographic and clinical data were obtained from medical records. All tissue analyzed and added to the study was processed in a comparable manner, the tumors were graded according to the modified Gleason grading system [26,27], and staged according to the World Health Organization guidelines [28]. All primary cancers were histologically reviewed by two pathologists (ER and LTB), and all demographic, clinical and histopathological data (Table 1) were recorded in an SPSS data file, in which patients were de-identified. The Regional Committee for Medical and Health Research Ethics (2009/1393), the Data Protection Official for Research (NSD), and the National Data Inspection Board approved this study. All patients were anonymized with each trial number. These numbers were initially linked to identity for only one purpose prior; to collect clinical information. The Norwegian Social Science Data Service and the University Hospital’s Data Protection Office accepted this solution (2009/1393). Written consent from the patients was considered, but as this was a retrospective study where most of the material was more than 10 years old and most of the patients deceased, it was considered not needed. All data was analysed anonymously.

**Table 1:** Patient characteristics and clinicopathological variables as predictors for BF, CF and PCD in PCa patients (n = 535) (univariate analyses; log rank test), significant p-values in bold (threshold p ≤ 0.05).

Characteristic	Patients (n)	Patients (%)	BF (170 events)		CF (36 events)		PCD (15 events)	
			5-yearEFS (%)	p	10-year EFS (%)	p	10-year EFS (%)	p
**Age**				0.55		0.085		0.600
≤ 65 years	357	67	76		92		97	
> 65 years	178	33	70		88		96	
**pT-Stage**				**<0.001**		**<0.001**		**0.027**
pT2	374	70	83		96		98	
pT3a	114	21	60		86		98	
pT3b	47	9	43		73		89	
**pN-stage**				**<0.001**		**<0.001**		**<0.001**
NX	264	49	79		95		98	
N0	268	50	71		89		97	
N1	3	1	0		33		67	
**Preop PSA**				**<0.001**		0.085		0.061
PSA < 10	308	58	80		93		99	
PSA > 10	221	41	67		88		95	
Missing	6	1	-		-		-	
**Gleason**				**<0.001**		**<0.001**		**0.001**
3+3	183	34	83		98		99	
3+4	220	41	76		94		98	
4+3	80	15	69		84		95	
≥ 4+4	52	10	45		71		89	
**Tumor size**				**<0.001**		**0.019**		0.098
0–20 mm	250	47	82		94		99	
> 20 mm	285	53	67		88		96	
**PNI**				**<0.001**		**<0.001**		**0.002**
No	401	75	79		95		98	
Yes	134	25	60		81		93	
**PSM**				**0.041**		**0.038**		0.697
No	249	47	81		94		97	
Yes	286	53	69		89		97	
**Non-apical PSM**				**<0.001**		**<0.001**		**0.029**
No	381	71	81		95		98	
Yes	154	29	57		81		94	
**Apical PSM**				**0.040**		0.484		0.313
No	325	61	73		90		96	
Yes	210	39	77		92		98	
**LVI**				**<0.001**		**<0.001**		**0.009**
No	492	92	77		93		98	
Yes	43	8	46		71		87	
**Surgical proc.**				0.230		0.414		0.581
Retropubic	435	81	76		90		97	
Perineal	100	19	67		95		98	

Abbreviations: BF = biochemical failure; CF = clinical failure; PCD = prostate cancer death; PCa = prostate cancer; EFS = event free survival in months; LVI = lymphovascular infiltration; NR = not reached; PNI = Perineural infiltration; Preop = preoperative; PSA = Prostate specific antigen; PSM = Positive surgical margin; Surgical proc = surgical procedure

### Microarray construction

Tissue Microarray (TMA) construction was chosen for high-throughput molecular pathology analyses. For each tissue block, a pathologist (ER) identified and marked two representative areas of epithelial tumor tissue and two for tumor stromal tissue on the corresponding haematoxylin and eosin slides. One area with normal epithelial cells, and one with normal stromal tissue were also carefully marked. From each of these areas, cores were sampled from each donor block in order to construct TMA blocks. Prostate cores from 20 patients without any history of malignancy were used as controls.

The TMAs were assembled using a tissue-arraying instrument (Beecher Instruments, Silver Springs, MD, USA). We used a 0.6 mm diameter needle to harvest cores from the marked tissue areas from each paraffin-embedded tissue blocks. The core samples were inserted into an empty recipient paraffin block in a precise array pattern. To include all core samples, twelve tissue array blocks were constructed. Multiple 4 μm sections were cut with a Micron microtome (HM355S), affixed to glass slides, and sealed with paraffin. The detailed methodology has been reported previously [29].

### Immunohistochemistry (IHC)

For immunohistochemical staining, the Ventana Benchmark XT automated staining system (Ventana Medical Systems, Tucson, AZ) and Ventana reagents were used. TMA slides were deparaffinised with xylene and rehydrated in decreasing concentrations of ethanol. Endogenous peroxidase was blocked using the Ventana endogenous peroxidase blocking kit after a rinse with distilled water. For antigen retrieval, slides were heated with cell conditioning solution (CC1, Ventana), standard, according to the manufacturer’s instructions. The following antibody from Ventana Medical (Tucson, Arizona, USA) was used in this study: CONFIRM anti-progesterone receptor (clone 1E2, catalogue # 790–4296) rabbit monoclonal primary antibody, directed against both A and B isoforms of the human progesterone receptor. The antibody was prediluted by the manufacturer. The applied antibody is produced for routine diagnostic IHC and has received FDA approval (510k) for IVD (*in vitro* diagnostic) use. The PGR antibody is currently applied in routine practice in assessment of PGR status in breast cancer. In order to validate specificity of the primary antibody against PGR, lysates of HEK 293 cells with either transiently overexpressed PGR or an empty vector were used. Further details regarding antibody validation can be found in the supporting information files (S1 Text, S2 Text, S1 Fig.). UltraView Universal DAB was used as detection kit. Finally, TMA slides were counterstained with haematoxylin to visualise the nuclei.

### Scoring of IHC

**Fig 1:**
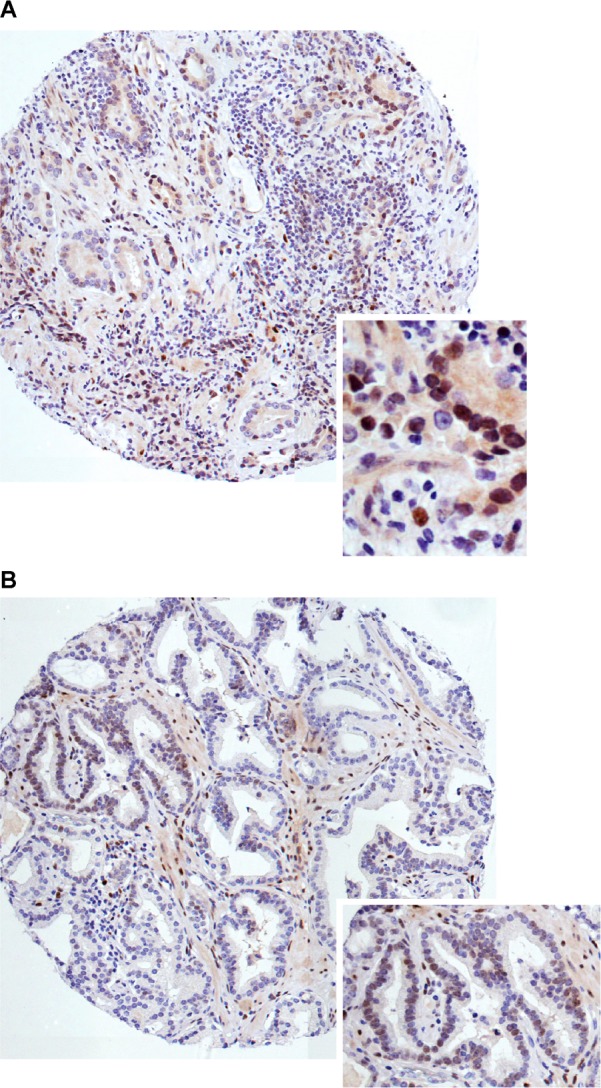
High and low progesterone receptor (PGR) protein density levels in human prostate cancer (PCa) tissue sections. Immunohistochemistry microscopic pictures of tissue micro array representing different expression of PGR staining in PCa sections A-B. (**A)** High density of PGR in tumor cells (TE), including magnification. **(B)** Low density of PGR in TE, including magnification. Original magnification x100 and x400.

The ARIOL imaging system (Applied Imaging Corp., San Jose, CA, USA) was used to scan and digitalise the IHC stained TMA slides. The slides were loaded in the SL 50 automated slide loader and scanned at a low resolution (1.25x) and high resolution (20x) using an Olympus BX61 microscope with an automated platform (Prior Scientific, Cambridge, UK). Images of the cores were uploaded into the Ariol Software. All samples were de-identified and scored manually by two pathologists (ER and SAS) independently of each other and both were blinded to any pathological or clinical information. In case of disagreement, the slides were re-examined and the observers reached a consensus. Representative viable tissue sections were scored semi-quantitatively and the degree of nuclear PRG expression by IHC was graded according to both dominant staining intensity and density in both TE and TS. Both intensity and density was given a score between 0–3. Intensity was scored as follows: 0 = negative, 1 = weak, 2 = moderate, 3 = strong. Density was scored according to the percentage of positive cells in the examined compartment using the following system: 0 = 0%, 1 = ≤ 5%, 2 = 5–50%, 3 = > 50%. For each case, mean scores were calculated. The mean scoring values were then connected to patient’s clinical and histopathological information. The scoring values were then dichotomised as high and low intensity or density of stained cells (Fig. 1) using optimal cut off values. In both TE and TS, cut off was defined as the density level × 4^th^ quartile. A high score was defined as density level ≥ 0.75 in TE, and ≥ 1.75 in TS.

### Statistical methods

All statistical analyses were performed using the statistical package IBM SPSS, version 21 (SPSS Inc., Chicago, IL, USA). The IHC scoring values from each pathologist were compared for inter-observer reliability by use of a two-way random effect model with absolute agreement definition. A Wilcoxon signed rank test was used to assess if there was statistically significant differences in PGR intensity and density between the different compartments of the PCa specimens. We employed the Spearman correlation coefficient to examine the association between PGR expression and clinopathological variables. The Kaplan-Meier method was used for the univariate survival analysis, and log-rank test to assess the statistical significance between the survival curves of the model. Univariate Kaplan Meier curves were constructed for the following the end-points: 1) Biochemical failure (BF), 2) Clinical failure (CF) and 3) PCa death (PCD). BF was determined as a PSA recurrence ≥ 0.4 ng/ml in a minimum of two different blood samples postoperatively [30]. CF was defined as verified local symptomatic progression beyond cure and/or findings of metastasis to bone, visceral organs or lymph nodes by CT, MR, bone scan or ultrasonography. PCD was defined as death caused by progressive and disseminated castration-resistant PCa uncontrollable by therapy. All significant variables from the univariate analysis were entered in the multivariate analysis using a backward stepwise Cox regression model with a probability for stepwise entry removal at 0.05 and 0.1, respectively. We considered a p-value < 0.05 as statistically significant for all analyses.

## Results

### Patient characteristics

The radical prostatectomy was retropubic in 435 cases and perineal in 100 cases. Patients’ age at surgery ranged from 47 to 76 years with a median age of 62 years. Further particulars regarding the cohort are previously published [31]. An overview of the demographic, clinical and histopathological characteristics is presented in Table 1. Combined Gleason score ranged from 6 to10 and tumor stage from T2a to T3b. At the last follow-up 170 (32%) experienced BF, 36 (7%) experienced CF and 15 (3%) had died due to PCa.

### Progesterone expression and correlation with clinicopathological variables

There was a good scoring agreement between the two investigating pathologists. The intra-class correlation coefficient (reliability coefficient, r) for the PGR marker was 0.78 (p < 0.001). PGR was expressed in the nucleus of both normal cells and in TE and TS (Fig. 1). High PGR density in TE (≥ 0.75) was found in 109 (20%) of the 535 patients whereas, a high PGR density (x 1.75) in TS was found in 120 (23%) of the patients. There was no significant difference in PGR density level in control epithelia compared to TE (p = 0.429), although the average density score was 0.37 in control epithelia and 0.52 in TE. In addition, 61.9% of the controls did not express PGR, while only 28.3% of TE were without PGR expression. However, there was a significantly higher PGR density level in TS compared to control stroma (p < 0.001). Further, a significantly higher expression intensity and density level of PGR was found in TS when compared to TE (both p < 0.001).

High density levels of PGR in TE were correlated with a positive apical margin (p = 0.025) and perineural infiltration (PNI) (p < 0.01). No correlations to other clinopathological variables were identified.

### Univariate analysis

**Fig 2:**
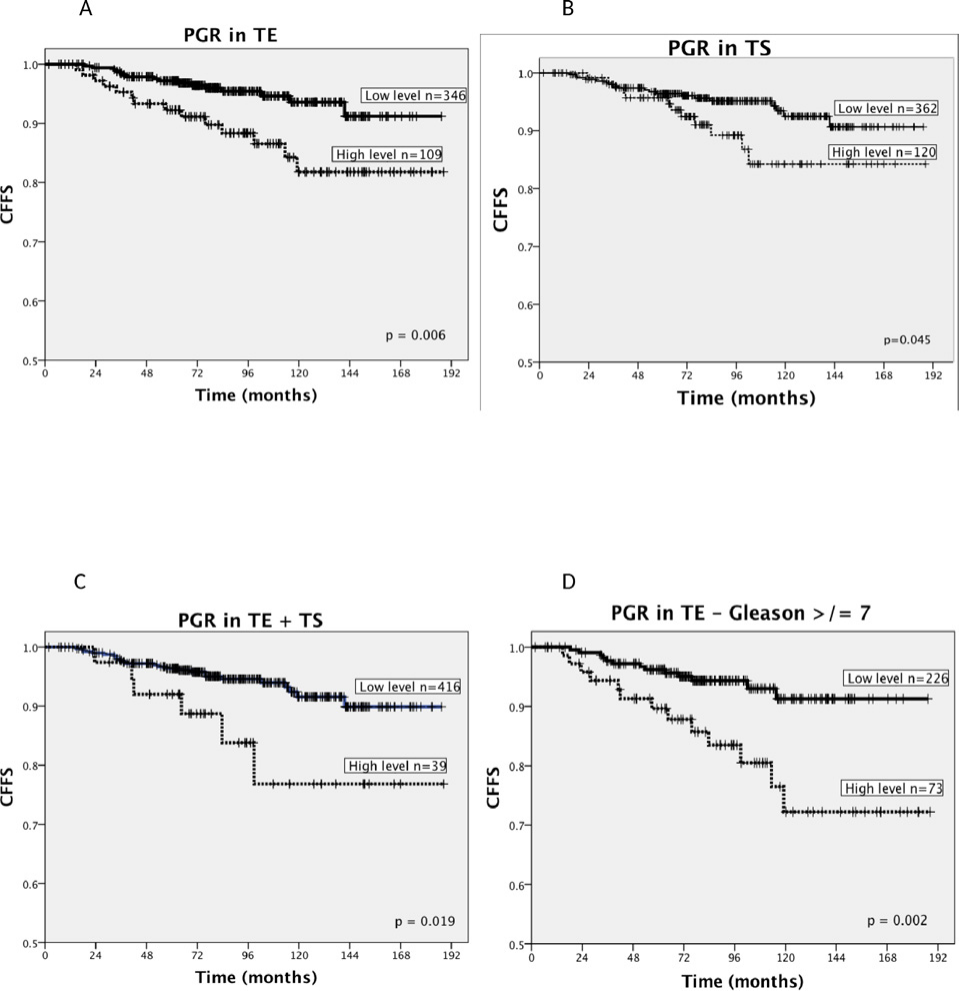
High progesterone receptor (PGR) density level is associated with reduced clinical failure free survival (CFFS). Kaplan-Meier curves displaying proportion of prostate caner patients (n = 535) with CFFS according to high and low density level of progesterone receptor (PGR) in different cell types A-D. **(A)** Tumor stromal cells (TS), **(B)** tumor cells (TE), **(C)** TE and TS combined and **(D)** TE in patients with a high Gleason score (≥ 7). A high PGR density level is significantly associated with a reduction in CFFS. In both TE and TS, cut off was defined as the density level × 4^th^ quartile. A high score was defined as density level ≥ 0.75 in TE, and ≥ 1.75 in TS. Log-rank tests were used to assess the statistical significance between the survival curves of the model. Median follow-up time was 89 (range 6–188) months. A p-value < 0.05 was considered statistically significant.

Associations between the density level of PGR and CFFS (clinical failure free survival) are presented in Table 2 and Fig. 2. The following clinopathological variables were all significant prognostic factors for CF: pT-stage (p < 0.001), pN-stage (p < 0.001), Gleason grade (p < 0.001), tumor size (p = 0.019), perineural infiltration (p = 0.001), positive surgical margin (p = 0.038), non-apical surgical margin (p < 0.001) and lymphovascular infiltration (p < 0.001) (Table 1).

**Table 2:** Expression of PGR in TE and TS as predictor for CFFS in PCa patients (n = 535), (univariate analysis; log rank test), significant p-values in bold (threshold p ≤ 0.05).

Marker expression	Patients (n)	Patients (%)	5-year CFFS (%)	10-year CFFS (%)	p
**PGR**					
**Density TE**					**0.006**
Low	346	65	97	94	
High	109	20	92	82	
Missing	80	15			
**Density TS**					**0.045**
Low	362	68	97	92	
High	120	22	96	84	
Missing	53	10			
**Density TE + TS**					**0.019**
Low	416	78	96	92	
High	39	7	92	77	
Missing	80	15			

Abbreviations: PGR = progesterone receptor; CFFS = clinical failure free survival; PCa = prostate cancer; TE = tumor epithelial cells; TS = tumor stromal cells

Increasing PGR density levels in both TE (p = 0.006) and TS (p = 0.045) were significantly associated with CF (Fig. 2, panel A and B). When merging PGR density levels in TE and TS, patients with high (high/high) PGR density levels had significantly reduced CFFS (p = 0.019) compared to those with low density levels (low/high, high/low and low/low) (Fig. 2, panel C). Ten year CFFS were 76.8% vs. 91.6% respectively for patients with high (high/high) density levels vs. low (low/high, high/low and low/low) (Table 2).

High PGR levels in TE showed a similar tendency for increased BF, but this was not statistically significant (p = 0.144). For TS or TS and TE combined, there were no associations with BF or PCD.

A high density level of PGR in TE was significantly associated with CF in the subgroup of patients with Gleason score ≥ 7 (p = 0.002, Fig. 2, panel D) compared to the subgroup of patients with Gleason score 6 (p = 0.914). Ten year CFFS for patients with high PGR density levels were 72.2% vs. 97.2% respectively for patients with Gleason score ≥ 7 vs. Gleason score 6.

### Multivariate analysis

In the multivariate analysis (Table 3), high expression of PGR in TE was an independent predictor for CF (HR: 2.5, 95% CI: 1.2–5.2, p = 0.012) in addition to Gleason grade (p = 0.001) and non-apical surgical margin (p = 0.006). PGR expression in TS tended to, but did not reach statistical significance (HR: 2.1, CI: 1.0–4.3, p = 0.060). No independent prognostic factors were found for BF and PCD.

**Table 3:** Cox regression analysis (backwards stepwise model) summarizing significant independent prognostic factors for CF in PCa patients (n = 535), significant p-values in bold (threshold p ≤ 0.05).

Factor	CF		
	HR	CI 95%	p
**Gleason**			0.001
3+3	1.00		
3+4	2.20	0.67–7.17	0.192
4+3	3.84	1.12–13.15	0.032
≥8	8.72	2.71–28.03	0.000
**PNI**			
No	NS		
Yes			
**Non-apical PSM**			0.006
No	1.00		
Yes	2.81	1.34–5.89	
**PGR TE**			0.012
Low	1.00		
High	2.51	1.23–5.17	

Abbreviations: PCa = prostate cancer; CF = clinical failure; PGR = progesterone receptor; TE = tumor epithelial cells; PNI = perineural infiltration; PSM = positive surgical margin

## Discussion

To our knowledge, this is the first large-scale study investigating the prognostic role of PGR in TE and TS in PCa. In univariate analysis, a high density level of PGR in both TE and TS was associated with CF. High density level of PGR in the TE was an independent prognostic factor for CF.

A first step to understand PGR action in PCa is to define receptor expression in prostate tissue. Previous publications on PGR expression in PCa, especially those using IHC, have presented contradicting results and only a few reports have addressed PGR’s role in prostate carcinogenesis. Our large-sized study demonstrates a wide distribution of PGR in stromal and epithelial cells of both benign and malignant prostate tissue. Currently, there seems to be a general agreement of PGR presence in the stromal cells of PCa [10,17,19–23]. In line with our findings, several have also reported a high PGR expression in TE of PCa [9,10,23,25]. In contrast, others have demonstrated a total lack of PGR expression in TE [17,19,20,22]. Even experimental studies using cell lines have reported conflicting results [17,24,25,32]. Such discrepancy may be explained by several factors. This includes use of different antibodies, tissue processing, antigen retrieval methods, number of tissue samples and different scoring systems, and may reflect a lack of methodological standardization. The monoclonal antibody 1E2 used in our study has been optimized for clinical use and is used in routine practice in assessment of PGR status in breast cancer. This is also the case for the NCL-PGR1A6 antibody applied in the articles by Bonkhoff et al. [10] and Hiramatsu et al. [23], which confirms the presence of PGR in TE. Several of the IHC studies contradicting the present study’s finding were performed prior to the development of new methods increasing methodological accuracy [17,19,20]. Such methods include the use of new, highly specific monoclonal antibodies against PGR, the microwave irradiation method and the time efficient tissue processing method TMA [33], which has been reported as a valuable tool for evaluation of patient material and a good substitute for whole section analysis [34].

Yu et al. recently investigated the location and role of both PGR isoforms in PCa and report findings contradictory to ours. This may be explained by their investigation of PGR which they found to be expressed solely in a subset of stromal cells of the 27 radical prostatectomy specimens [22]. This is in contrast to our work were the expression of PGR in both TE and TS was clearly detected. In our cohort 129 (28.3%) patients had no PGR expression in TE, in contrast to only 8 patients (1.7%) with negative PGR staining in TS. However, those with a high density level of PGR in TE was significantly associated with CF. The difference in cohort size could potentially explain some of the discrepancy between the findings. In addition, both the chosen antibody and tissue processing methods differ.

In another study using cell proliferation assay, Yu et al. found PGR to be negatively regulating stromal cell proliferation *in vitro* [32]. In our work univariate analysis demonstrated a high PGR expression in TS to be associated with clinical failure in PCa patients. So far we have not yet demonstrated the mechanism underlying this association.

Steroid hormones regulate the cell’s progression through cell cycle by binding to their respective receptors. These receptors are signal transduction molecules and can regulate the proliferation in two different ways, genomic or non-genomic actions in complex signalling networks [6]. Several non-genomic proliferative actions of progesterone have been proposed in tumor cells of other organs, including breast [35–37], astrocytoma [38] and osteosarcoma [39] cell lines. However, such results are contradicted by suggestions of anti-proliferative actions of progesterone in endometrial cancer [40]. This could indicate that the actions of progesterone are tissue specific. We found that high PGR density level in TE was associated with CF in patients with Gleason score ≥ 7, suggesting an up-regulation of the PGR in progressing PCa. This is in consistence with previous publications. Bonkhoff et al. have suggested progressive emergence of PGR during PCa progression and metastasis [10]. Supporting these findings, Latil and co-workers found a decreased PGR expression in clinically localized tumors and increased PGR expression in hormone-refractory tumors, when compared with normal prostate tissue [9].

In several experimental studies by Check et al., mice with PCa were treated with a PGR antagonist, mifepristone, and compared with controls. They found a higher mortality in those not treated. Moreover, there were less PCa complications in the treated group [41,42]. Similar findings of anti-progesterone activity of mifepristone in both androgen sensitive and non-sensitive PCa cell lines *in vitro* and *in vivo*, have been reported [43,44]. Our findings provide further support to these findings, indicating that PGR plays a role in the pathogenesis of PCa. To investigate whether aberrant PGR activity is a mechanism of castrate resistant prostate cancer development, a phase I/II clinical trial has just been initiated to test the effect of the anti-progestin, onapristone, in patients with this condition (http://clinicaltrials.gov/show/NCT02049190).

The mechanism behind the PGR up-regulation in PCa has not yet been elucidated. In this study, Ki67 and PGR in TE were correlated with CF (S3 Text), indicating an association between PGR and proliferative activity. Arora et. al. [12] have reported that up-regulation of the glucocorticoid receptor re-activates the expression of a subset of androgen receptor-regulated genes and thereby induces castrate resistant PCa. The PGR is, like the glucocorticoid receptor, similar to androgen receptor with 88% sequence homology in the ligand-binding domain [45]. In line with this finding, progesterone induced expression of androgen receptor-regulated genes could be a potential mechanism contributing to the development of castrate resistant PCa. However, further research investigating this is warranted for such a hypothesis to be confirmed.

A possibility of different roles by the two PGR isoforms in normal prostate tissue and PCa, as is suggested for the estrogen receptors [13], must also be taken into account. We now know that crosstalk between TE and TS is essential for the development of PCa. In a study by Memarzadeh et al., cancer-associated fibroblast growth factors caused an up-regulation of epithelial androgen receptor [46]. This could indicate that epithelial-stromal crosstalk is the mechanism behind induction of PGR expression in TE and it is thereby promoting PCa progression. However, up-regulation of PGR may not be the direct mechanism behind increased proliferation, but rather a consequence of other underlying processes. Thus, the respective role of epithelial versus stromal PGR in prostate carcinogenesis and a potential individual role of the PGR isoforms remain to be determined.

## Conclusion

Herein, we found that a high density level of PGR in TE is an independent prognostic factor for progression to CF in PCa. Further, high PGR density levels are significant for progression to CF in patients with Gleason score ≥ 7. Progesterone/PGR may for these reasons be useful as a prognostic tool, but also as a target for novel treatment strategies in PCa. Further functional studies investigating PGR’s role in both epithelial and stromal compartments of PCa are still needed to conclude how to best apply this knowledge in PCa diagnosis and treatment.

## Supporting Information

S1 Dataset SPSS PCa and PGR dataset. (SAV)

S2 Dataset SPSS PCa and Ki67 dataset. (SAV)

S1 Fig Lysates of HEK 293 cells with either an empty vector (A) or a PGR overexpression construct (B) were ran on a SDS-PAGE gel and transferred onto a nitrocellulose membrane. The membrane was first probed with the Ventana anti-PGR antibody (upper panel), and then with the anti-actin antibody to control for loading (lower panel).
(TIFF)

S1 Text Antibody specificity validation. (DOCX)

S2 Text Ventana product catalogue, CONFIRM anti-Progesterone Receptor (PR) (1E2) Rabbit Monoclonal Primary Antibody. (PDF)

S3 Text Ki67 immunostaining. (DOCX)
