# Supplementary figures and images for: High Progesterone Receptor Expression in Prostate Cancer Is Associated with Clinical Failure
Source: PLoS One. 2015 Feb 27;10(2):e0116691. doi: 10.1371/journal.pone.0116691 (PMC4344236; doi:10.1371/journal.pone.0116691)

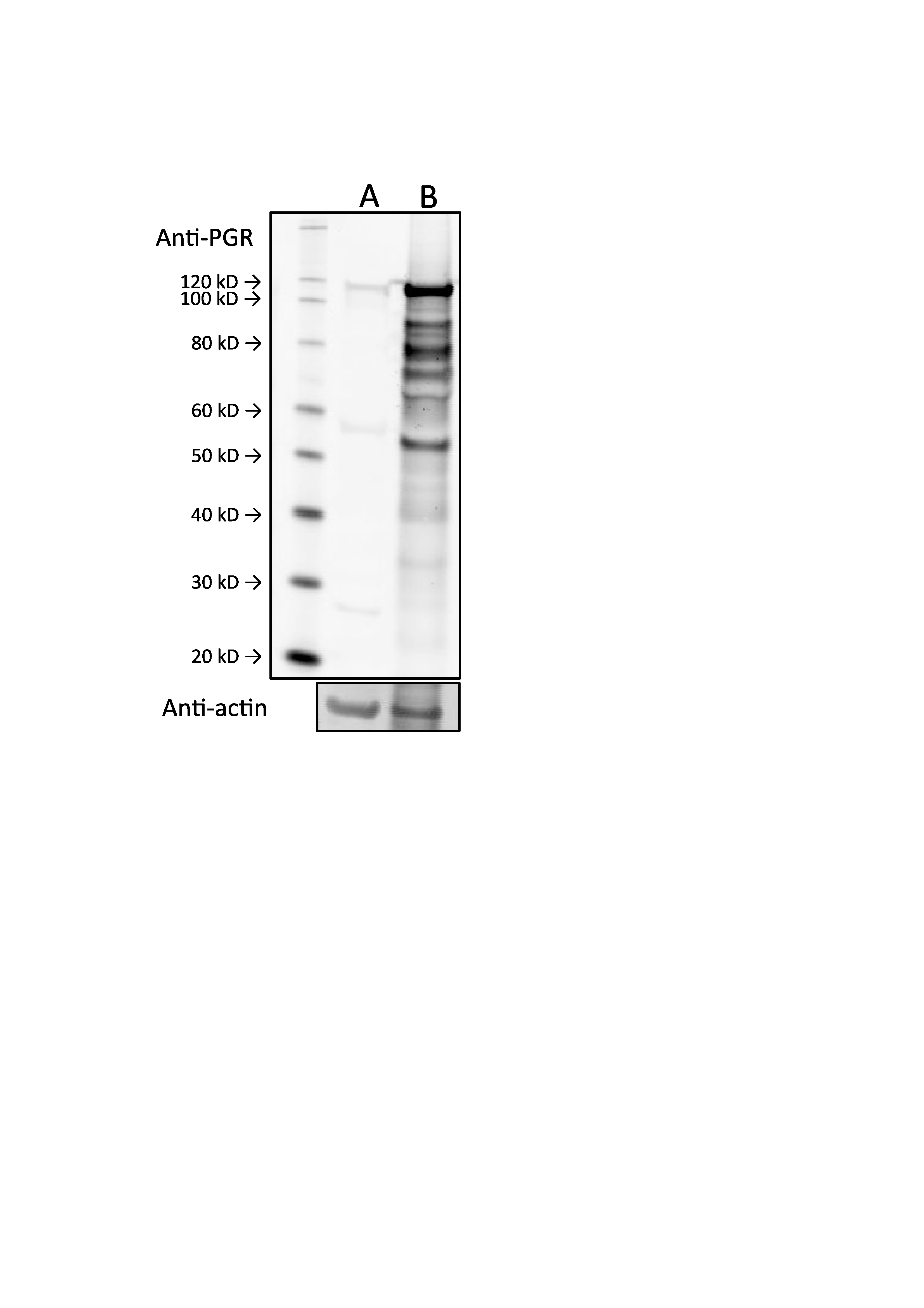

Supplement: S1 Fig — The membrane was first probed with the Ventana anti-PGR antibody (upper panel), and then with the anti-actin antibody to control for loading (lower panel). (TIFF) [file pone.0116691.s003.tiff]
