# Supplementary material for: High Progesterone Receptor Expression in Prostate Cancer Is Associated with Clinical Failure
Source: PLoS One. 2015 Feb 27;10(2):e0116691. doi: 10.1371/journal.pone.0116691 (PMC4344236; doi:10.1371/journal.pone.0116691)
Supplement: S1 Text — (DOCX) [file pone.0116691.s004.docx]

**Antibody specificity validation**

**Methods**

HEK 293 cell lysates were acquired from OriGene (cat# LY424456) and were incubated with 2xSDS Sample Buffer (4% SDS, 125mM Tris-HCl pH6.8, 10% Glycerol, 0.002% Bromphenol blue, 100mM DTT) for 10 minutes at 100°C, and run on a NuPAGE 4–12% Bis Tris Gel (Life Technologies, USA). Blotting was performed onto an Odyssey nitrocellulose membrane (LI-COR Biosciences, Germany) using the NuPAGE blotting system (Life Technologies, USA). The membrane was incubated with Odyssey blocking buffer (LI-COR Biosciences) for 1 hour at room temperature. Primary and secondary antibodies were diluted in the blocking buffer. CONFIRM Anti-PGR antibody (Ventana Medical Systems, USA, cat# 790-4296, clone 1E2) was used in the dilution of 1:5000, and anti-actin antibody (Sigma, cat#A2066) 1:1000. IRDye CW secondary antibodies (LI-COR, Germany) were used in dilution 1:10000. Molecular weight markers used were SeeBlue Plus 2 (Life Technologies, USA, cat#LC5925) and Magic Mark XP (Life Technologies, USA, cat#LC5602). Images were acquired on the ODYSSEY Sa Infrared Imaging System (LI-COR, Germany).

**Results**

In order to validate specificity of the primary antibody against PGR, we used lysates of HEK 293 cells with either transiently overexpressed PGR or an empty vector (Figure S1). According to the manufacturer of the anti-PGR antibody, it should recognize bands of sizes 60, 87 and 110 kD, which is close to what we observe on the Western blot. Minor variations in size can be explained by post-translational modifications and specific conditions of sample preparation.

**Figure legend**

**Figure S1**

Lysates of HEK 293 cells with either an empty vector (A) or a PGR overexpression construct (B) were ran on a SDS-PAGE gel and transferred onto a nitrocellulose membrane. The membrane was first probed with the Ventana anti-PGR antibody (upper panel), and then with the anti-actin antibody to control for loading (lower panel).
