# Supplementary material for: High Progesterone Receptor Expression in Prostate Cancer Is Associated with Clinical Failure
Source: PLoS One. 2015 Feb 27;10(2):e0116691. doi: 10.1371/journal.pone.0116691 (PMC4344236; doi:10.1371/journal.pone.0116691)
Supplement: S2 Text — (PDF) [file pone.0116691.s005.pdf]

CONFIRM anti-Progesterone Receptor (PR) (1E2) Rabbit Monoclonal Primary Antibody

REF 790-2223 05277990001 Σ 50

790-4296 05278392001 Σ 250

IVD

INTENDED USE

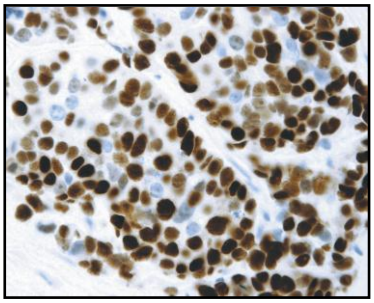

Figure 1. CONFIRM anti Progesterone Receptor (PR) (1E2) staining of breast ductal carcinoma.

This antibody is intended for *in vitro* diagnostic (IVD) use.

CONFIRM anti-Progesterone Receptor (PR) (1E2) Rabbit Monoclonal (IgG) is intended for laboratory use for the qualitative detection of progesterone receptor (PR) antigen in sections of formalin-fixed, paraffin-embedded tissue on a VENTANA automated slide stainer with VENTANA detection kits and ancillary reagents. CONFIRM anti-PR (1E2) is directed against an epitope present on human progesterone receptor protein located in the nucleus of PR positive normal and neoplastic cells. CONFIRM anti-PR (1E2) is indicated as an aid in the management, prognosis, and prediction of therapy outcome of breast carcinoma.

This product should be interpreted by a qualified pathologist in conjunction with histological examination, relevant clinical information, and proper controls.

Prescription use only.

SUMMARY AND EXPLANATION

CONFIRM anti-PR (1E2) is a rabbit monoclonal antibody that recognizes the A and B forms of human progesterone receptor. The immunogen was developed from a synthetic peptide identified as an area of potential high antigenicity common to progesterone receptor A and B forms. The peptide was synthesized and covalently bound to keyhole limpet hemocyanin to further increase antigenicity. CONFIRM anti-PR (1E2) has been shown to react with 60 kD, 87 kD and 110 kD proteins from T47D cells via Western blotting. The protein sizes are in agreement with the predicted molecular weight of progesterone receptor forms A, B and C.[1,2]

Rabbit monoclonal antibodies have demonstrated improved sensitivity and specificity in immunohistochemistry.[3] Their reliability and staining quality is well established in breast carcinoma cases.[4,5] In one study, CONFIRM anti-PR (1E2) was used to determine semiquantitative hormone receptor values using a modified H-score based on percentage and intensity of staining. Subsequent quantitative measurement of PR receptors using RT-PCR in 80 breast cases demonstrated linear concordance when compared to CONFIRM anti-PR (1E2).[6]

Staining results with CONFIRM anti-PR (1E2) in normal tissues, neoplastic tissues, and 173 cases of breast carcinoma were evaluated by Ventana. In the 90 normal tissues tested, expression was consistent with the published literature in that PR was localized to the nucleus, and expression was limited to reproductive tissues (breast, cervix, and uterus), pancreatic islets, anterior pituitary and thyroid.[7-12]

Breast cancer is the most common carcinoma occurring in women, and the second leading cause of cancer related death.[11] Early detection and appropriate treatment therapies can significantly affect overall survival.[13,14] Small tissue samples may be easily used in routine immunohistochemistry (IHC), making this technique, in combination with antibodies that detect antigens important for carcinoma interpretation, an effective tool for the pathologist in diagnosis and prognosis of disease. An important marker in breast cancer today is progesterone receptor (PR), due to its role in determining the functionality of estrogen receptors present in breast cancer cells.

The presence of estrogen receptor (ER), however, does not guarantee a response to endocrine therapy. Half of all patients with ER positive primary tumors fail to respond.[15,16] In tumor cells, genetic expression is often inaccurate, leading to variant protein expression. It is proposed that mutant ER, that no longer bind estrogen or that no longer perform signal transduction, may be the cause of the failure to respond to endocrine therapy. One way to evaluate the functionality of the ER present in a breast carcinoma is to determine if proteins regulated by ER are expressed. Progesterone receptor is such a protein, and has been used to monitor for functional ER for many years.[17]

Determination of ER status for all primary breast carcinomas was recommended by the National Institutes of Health (NIH) in 1979, to better determine appropriate therapy. In 1985, both the NIH and the American Cancer Society independently published reports in support of determining hormone receptor status as an aid in the management of breast cancer. In 1996, the American Society of Clinical Oncology, based upon the findings of their Tumor Marker Panel, recommended the determination of ER and PR status on all primary lesions and on metastases if the results would influence treatment planning. They also recommended using the ER and PR results for identification of patients most likely to benefit from endocrine adjuvant therapy and therapy for recurrent or metastatic disease. The Tumor Marker Panel also noted that ER and PR status may play a role in prognosis, but cautioned that they should be evaluated in conjunction with other clinical criteria, as ER and PR status alone were relatively weak predictors of long term relapse and breast cancer related mortality.[18] In 2010, the American Society of Clinical Oncology and the College of American Pathologists (CAP) published a *Guideline for Immunohistochemical Testing of Estrogen and Progesterone Receptors in Breast Cancer* and recommends that ER and PR status be determined on all invasive breast cancers and breast cancer recurrences.[19]

A number of methodologies to assess PR status have been in use. FDA approved therapies have included cytosol receptor assay (SBA/DCC), analyzed by Scatchard plot, histochemical analysis of tissue using fluorescent microscopy, and enzyme immunoassay (EIA) using Anti-PR monoclonal antibody conjugate.[20]

Interpretation of the results of any detection system for PR must take into consideration the heterogeneity of breast cancer tumors. The tumors frequently contain benign epithelial cells from normal hyperplastic lobules or ducts that are also positive for PR. Thus, tests utilizing tissue homogenates such as DCC or EIA may not be solely a reflection of PR status in malignant tissue.[18] Histological tissue preparations have the advantage of intact tissue morphology to aid in the interpretation of the PR positivity of the sample. All histological tests should be interpreted by a specialist in breast cancer morphology or pathology, and the results should be used in conjunction with other clinical and laboratory data.

REAGENT PROVIDED

**Catalog Number 790-2223**	CONFIRM anti-PR (1E2) contains sufficient reagent for 50 tests.
	One 5 mL dispenser CONFIRM anti-PR (1E2) contains approximately 5 µg of a rabbit monoclonal antibody directed against human PR antigen.
**Catalog Number 790-4296**	CONFIRM anti-PR (1E2) contains sufficient reagent for 250 tests.
	One 25 mL dispenser CONFIRM anti-PR (1E2) contains approximately 25 µg of a rabbit monoclonal antibody directed against human PR antigen.

The antibody is diluted in 0.05 M Tris-HCl with 2% carrier protein, and 0.1% ProClin 300, a preservative. There is trace (~0.2%) fetal calf serum of U.S. origin from the stock solution.

Total protein concentration of the reagent is approximately 10 mg/mL. Specific antibody concentration is approximately 1 µg/mL. There is no known non-specific antibody reactivity observed in this product.

CONFIRM anti-PR (1E2) is a rabbit monoclonal antibody produced as a cell culture supernatant.

MATERIALS REQUIRED BUT NOT PROVIDED

Staining reagents such as VENTANA detection kits (i.e., *ultra*View Universal DAB Detection Kit) and ancillary components, including negative and positive tissue control slides, are not provided.

STORAGE

Store at 2-8°C. Do not freeze.

To ensure proper reagent delivery and the stability of the antibody, replace the dispenser cap after every use and immediately place the dispenser in the refrigerator in an upright position.

Every antibody dispenser is expiration dated. When properly stored, the reagent is stable to the date indicated on the label. Do not use reagent beyond the expiration date.

SPECIMEN PREPARATION

Routinely processed, formalin-fixed, paraffin-embedded tissues are suitable for use with this primary antibody when used with VENTANA detection kits and the VENTANA BenchMark XT automated slide stainer or the VENTANA BenchMark ULTRA automated slide stainer. The following steps are recommended for processing of specimens:[21]

1. Place specimen in 10% neutral buffered formalin. The amount used is 15 to 20 times the volume of tissue. No fixative will penetrate more than 2 to 3 mm of solid tissue or 5 mm of porous tissue in a 24 hour period. A 3 mm or smaller section of tissue should be fixed no less than 4 hours and no more than 8 hours. Fixation can be performed at room temperature (15-25°C).
2. After fixation specimen is placed in a tissue processing instrument for overnight preparation. Briefly, this processing consists of dehydration of specimen with alcohols followed by clearing reagents to remove alcohols and finally infiltration with paraffin.
3. Samples are embedded with paraffin in tissue cassettes and approximately 4 μm thick sections are cut, centered and picked up on glass slides. The slides should be Superfrost Plus or equivalent. Tissue should be air dried by placing the slides at ambient temperature overnight or placed in a 60°C oven for 30 minutes.
4. Slides should be stained promptly, as antigenicity of cut tissue sections may diminish over time.

It is recommended that positive and negative tissue controls be run simultaneously with unknown specimens.

WARNINGS AND PRECAUTIONS

1. This product contains 1% or less bovine serum, which is used in the manufacture of the antibody.
2. Avoid contact of reagents with eyes and mucous membranes. If reagents come in contact with sensitive areas, wash with copious amounts of water.
3. Avoid microbial contamination of reagents.
4. ProClin 300 is used as a preservative in this solution. It is classified as an irritant and may cause sensitization through skin contact. Take reasonable precautions when handling. Avoid contact of reagents with eyes, skin, and mucous membranes. Use protective clothing and gloves.
5. Consult local or state authorities with regard to recommended method of disposal.
6. Refer to product Safety Data Sheet for additional information.

PRINCIPLES AND PROCEDURES

CONFIRM anti-PR (1E2) binds to PR in paraffin embedded tissue sections. The specific antibody can be localized by either a biotin conjugated secondary antibody formulation that recognizes rabbit immunoglobulins, followed by the addition of a streptavidin horseradish peroxidase (HRP) conjugate (*i*VIEW DAB Detection Kit) or a secondary antibody-HRP conjugate (*ultra*View Universal DAB Detection Kit). The specific antibody-enzyme complex is then visualized with a precipitating enzyme reaction product.

Clinical cases should be evaluated within the context of the performance of appropriate controls. Ventana Medical Systems, Inc. (Ventana) recommends the inclusion of a positive tissue control fixed and processed in the same manner as the patient specimen (for example, a weakly positive breast carcinoma or uterus). In addition to staining with CONFIRM anti-PR (1E2), a second slide should be stained with VENTANA CONFIRM Negative Control Rabbit Ig. For the test to be considered valid, the positive control tissue should exhibit nuclear staining of the tumor cells or uterine glands and stroma. These components should be negative when stained with CONFIRM Negative Control Rabbit Ig. In addition, it is recommended that a negative tissue control slide (for example, a PR negative breast carcinoma) be included for every batch of samples processed and run on the VENTANA automated slide stainer. This negative tissue control should be stained with CONFIRM anti-PR (1E2) to ensure that the antigen enhancement and other pretreatment procedures did not create false positive staining.

Staining Procedure

VENTANA primary antibodies have been developed for use on a VENTANA automated slide stainer in combination with VENTANA detection kits and accessories. Recommended staining protocols for the automated slide stainers are listed in Table 1 and Table 2. The parameters for the automated procedures can be displayed, printed and edited according to the procedure in the instrument's Operator's Manual. Refer to the appropriate VENTANA detection kit package insert for more details regarding immunohistochemistry staining procedures.

Verification and validation of the recommended staining procedure for each detection kit is demonstrated through design control testing and results of clinical studies.

Any modification to the recommended staining procedure nullifies the Performance Characteristics provided in this package insert. The user must validate any modification to the recommended staining procedure.

Table 1. Recommended Staining Protocols for CONFIRM anti-PR (1E2) using *ultra*View Universal DAB Detection Kit on a BenchMark XT and BenchMark ULTRA Instrument.

	Instrument/Method	
**Procedure Type**	**BenchMark XT Instrument**	**BenchMark ULTRA Instrument**
Deparaffinization	Selected	Selected
Cell Conditioning (Antigen Unmasking)	Cell Conditioning 1, Standard	Cell Conditioning 1, Standard
Enzyme (Protease)	None required	None required
Antibody (Primary)	16 minutes, 37°C	16 minutes, 36°C
A/B Block (Biotin Blocking)	N/A	N/A
Counterstain (Hematoxylin)	Hematoxylin II, 4 minutes	Hematoxylin II, 4 minutes
Post Counterstain	Bluing, 4 minutes	Bluing, 4 minutes

Table 2. Recommended Staining Protocols for CONFIRM anti-PR (1E2) using *i*VIEW DAB Detection Kit on a BenchMark XT and BenchMark ULTRA Instrument.

	Instrument/Method	
**Procedure Type**	**BenchMark XT Instrument**	**BenchMark ULTRA Instrument**
Deparaffinization	Selected	Selected
Cell Conditioning (Antigen Unmasking)	Cell Conditioning 1, Standard	Cell Conditioning 1, Standard
Enzyme (Protease)	None required	None required
Antibody (Primary)	16 minutes, 37°C	16 minutes, 36°C
A/B Block (Biotin Blocking)	Required	Required
Counterstain (Hematoxylin)	Hematoxylin II, 4 minutes	Hematoxylin II, 4 minutes
Post Counterstain	Bluing, 4 minutes	Bluing, 4 minutes

The procedures for staining on the VENTANA automated slide stainers are as follows. For more detailed instructions and additional protocol options, refer to your Operator's Manual.

BenchMark Automated IHC/ISH Slide Stainers

1. Apply slide barcode label that corresponds to the antibody protocol to be performed.

2. Load the primary antibody, appropriate detection kit dispensers and required accessory reagents onto the reagent tray and place the reagent tray on the automated slide stainer.
3. Check bulk fluids and waste.
4. Load the slides onto the automated slide stainer.
5. Start the staining run.
6. At the completion of the run, remove the slides from the automated slide stainer.
7. Wash in a mild dishwashing detergent or alcohol to remove the coverslip solution.
8. Dehydrate, clear, and coverslip with permanent mounting media in the usual manner.

QUALITY CONTROL PROCEDURES

Positive Tissue Control

A positive tissue control must be run with every staining procedure performed. CAP recommends that a positive tissue control should be on the patient slide.[19] An example of tissue to use as a positive control with CONFIRM anti-PR (1E2) is a weakly positive breast carcinoma. The positive staining cells or tissue components (nuclear staining of tumor cells) are used to confirm that CONFIRM anti-PR (1E2) was applied and the instrument functioned properly. This tissue may contain both positive and negative staining cells or tissue components and serve as both the positive and negative control tissue. Control tissues should be fresh autopsy, biopsy or surgical specimens prepared or fixed as soon as possible in a manner identical to the test sections. Such tissues may monitor all steps of the procedure, from tissue preparation through staining. Use of a tissue section fixed or processed differently from the test specimen will provide control for all reagents and method steps except fixation and tissue processing.

A tissue with weak positive staining is more suitable than strong positive staining for optimal quality control and for detecting minor levels of reagent degradation. Ideally, a breast carcinoma tissue, which is known to have weak but positive staining, should be chosen to ensure that the system is sensitive to small amounts of reagent degradation or problems with the IHC methodology.

Alternatively, normal human proliferative endometrium may be used for a positive control. The positive staining components are nuclear staining of the glandular epithelia, and stromal and smooth muscle cells. Endometrial tissue, however, may not stain weakly enough to detect small amounts of reagent degradation or problems with the IHC methodology.

Known positive tissue controls should be utilized only for monitoring the correct performance of processed tissues and test reagents, and not as an aid in determining a specific diagnosis of patient samples. If the positive tissue controls fail to demonstrate positive staining, results with the test specimens should be considered invalid.

Negative Tissue Control

Use a tissue control known to be fixed, processed and embedded in a manner identical to the patient sample(s) with each staining run to verify the specificity of CONFIRM anti-PR (1E2) for demonstration of PR, and to provide an indication of specific background staining (false positive staining). Also the variety of different cell types in most tissue sections can be used by the laboratorian as internal negative control to verify CONFIRM anti-PR (1E2) performance specifications. For example, the same tissue (endometrium) used for the positive tissue control may be used as the negative tissue control. The components that do not stain (cytoplasm, cell membrane) should show absence of specific staining in cells not expected to stain, and provide an indication of specific background staining. The negative tissue control also should be used as an aid in interpretation of results. The variety of different cell types present in most tissue sections frequently offers negative control sites, but this should be verified by the user. If specific staining occurs in the negative tissue control sites, results with the patient specimens should be considered invalid.

Negative Reagent Control

A negative reagent control must be run for every specimen to aid in the interpretation of results. A negative reagent control is used in place of the primary antibody to evaluate nonspecific staining and allow better interpretation of specific staining at the antigen site. This provides an indication of nonspecific background staining for each slide. In place of the primary antibody, stain the slide with CONFIRM Negative Control Rabbit Ig, a purified non-immune rabbit IgG not reacting with human specimens. If an alternative negative reagent control is used, dilute to the same dilution as the primary antibody antiserum with VENTANA Antibody Diluent. Approximately 0.2% fetal calf serum is retained in the CONFIRM anti-PR (1E2). Addition of 0.2% fetal calf serum in VENTANA Antibody Diluent is also suitable for use as a nonspecific negative reagent control. The incubation period for the negative reagent control should equal the primary antibody.

When panels of several antibodies are used on serial sections, a negative reagent control on one slide may serve as a negative or nonspecific binding background control for other antibodies.

Assay Verification

Prior to initial use of this antibody in a diagnostic procedure, or if there is a change of lot number, the specificity of the antibody should be verified by staining a number of positive and negative tissues with known performance characteristics. Refer to the quality control procedures previously outlined in this section of the product insert and to the quality control recommendations of the College of American Pathologists Laboratory Accreditation Program, Anatomic Pathology Checklist, or the CLSI Approved Guideline or both documents.[22,23] These quality control procedures should be repeated for each new antibody lot or whenever there is a change of lot number of one of the reagents in a matched set or a change in assay parameters. Quality control cannot be meaningfully performed on an individual reagent in isolation since the matched reagents, along with a defined assay protocol, must be tested in unison before using a kit for diagnostic purposes. Tissues listed in the Summary of Expected Results are suitable for assay verification.

All quality control requirements should be performed in conformance with local, state and federal regulations or accreditation requirements.

STAINING INTERPRETATION

The VENTANA automated immunostaining procedure causes a colored reaction product to precipitate at the antigen sites localized by CONFIRM anti-PR (1E2). A qualified pathologist experienced in immunohistochemistry procedures must evaluate positive and negative controls and qualify the stained product before interpreting results. Progesterone receptor status is determined by the percentage of stained tumor cells. A case is considered PR positive if there is staining of the nucleus in equal to or greater than 1% of tumor cells.

Positive Tissue Control

The positive tissue control stained with CONFIRM anti-PR (1E2) should be examined first to ascertain that all reagents are functioning properly. The presence of a brown (3,3" diaminobenzidine tetrachloride, DAB) reaction product within the target cells' nuclei is indicative of positive reactivity. An example of a tissue that may be used as a positive control is a known weakly positive breast carcinoma, e.g. $\geq$1%. Nuclei of the tumor cells should be positive, with the stroma remaining PR negative. It is imperative that only nuclear staining be considered positive if a false positive interpretation is to be avoided. Normal human endometrium may also be used. In normal endometrium, PR staining is seen in nuclei of the endometrial glands and stroma. If the positive tissue controls fail to demonstrate appropriate positive staining, any results with the test specimens should be considered invalid.

Negative Tissue Control

The negative tissue control should be examined after the positive tissue control to verify the specific labeling of the target antigen by the primary antibody. The absence of specific staining in the negative tissue control confirms the lack of antibody cross reactivity to cells or cellular components. The breast carcinoma used as a positive control may also be used as a negative control tissue. Stromal elements should show no nuclear staining. If specific staining occurs in the negative tissue control, results with the patient specimen should be considered invalid.

Nonspecific staining, if present, will have a diffuse appearance. Sporadic light staining of connective tissue may also be observed in tissue sections that are excessively formalin fixed. Intact cells should be used for interpretation of staining results, as necrotic or degenerated cells will often stain nonspecifically.[24]

Patient Tissue

Patient specimens stained with CONFIRM anti-PR (1E2) should be examined last. Positive staining intensity should be assessed within the context of any nonspecific background staining of the negative reagent control. PR may be detected among other neoplasms, such as cancers of the ovary and endometrium.[10] The morphology of each tissue sample should also be examined utilizing a hematoxylin and eosin stained section when interpreting any immunohistochemical result. The patient's morphologic findings and pertinent clinical data must be interpreted by a qualified pathologist. Refer to Summary and Explanation, Limitations, and Summary of Expected Results for specific information regarding immunoreactivity.

LIMITATIONS

General Limitations

1. Immunohistochemistry is a multiple step diagnostic process that requires specialized training in the selection of the appropriate reagents and tissues, fixation, processing, preparation of the immunohistochemistry slide, and interpretation of the staining results.
2. Tissue staining is dependent on the handling and processing of the tissue prior to staining. Improper fixation, freezing, thawing, washing, drying, heating, sectioning, or contamination with other tissues or fluids may produce artifacts, antibody trapping, or false negative results. Inconsistent results may be a consequence of variations in fixation and embedding methods, or from inherent irregularities within the tissue.
3. Excessive or incomplete counterstaining may compromise proper interpretation of results.
4. The clinical interpretation of any positive staining, or its absence, must be evaluated within the context of clinical history, morphology and other histopathological criteria. The clinical interpretation of any staining, or its absence, must be complemented by morphological studies and proper controls as well as other diagnostic tests. This antibody is intended to be used in a panel of antibodies. It is the responsibility of a qualified pathologist to be familiar with the antibodies, reagents and methods used to produce the stained preparation. Staining must be performed in a certified, licensed laboratory under the supervision of a pathologist who is responsible for reviewing the stained slides and assuring the adequacy of positive and negative controls.
5. Ventana provides antibodies and reagents at optimal dilution for use when the provided instructions are followed. Any deviation from recommended test procedures may invalidate expected results. Appropriate controls must be employed and documented. Users who deviate from recommended test procedures must accept responsibility for interpretation of patient results.
6. This product is not intended for use in flow cytometry, performance characteristics have not been determined.
7. Reagents may demonstrate unexpected reactions in previously untested tissues. The possibility of unexpected reactions even in tested tissue groups cannot be completely eliminated because of biological variability of antigen expression in neoplasms, or other pathological tissues.[25] Contact your local support representative with documented unexpected reactions.
8. Tissues from persons infected with hepatitis B virus and containing hepatitis B surface antigen (HBsAg) may exhibit nonspecific staining with horseradish peroxidase.[26]
9. When used in blocking steps, normal sera from the same animal source as the secondary antisera may cause false negative or false positive results due to autoantibodies or natural antibodies.
10. False positive results may be seen because of nonimmunological binding of proteins or substrate reaction products. They may also be caused by pseudoperoxidase activity (erythrocytes), endogenous peroxidase activity (cytochrome C), or endogenous biotin (example: liver, brain, breast, kidney) depending on the type of immunostain used.[24]
11. As with any immunohistochemistry test, a negative result means that the antigen was not detected, not that the antigen was absent in the cells or tissue assayed.

Specific Limitations

1. The antibody, in combination with VENTANA detection kits and accessories, detects antigen that survives routine formalin fixation, tissue processing and sectioning. Users who deviate from recommended test procedures are responsible for interpretation and validation of patient results.
2. A CONFIRM anti-PR (1E2) negative result does not exclude the presence of PR. Negative reactions in breast carcinomas may be due to loss or marked decrease of expression of antigen. Therefore, it is recommended that this antibody be used in a panel of antibodies including estrogen receptor.
3. This antibody is not intended for use in manual staining procedures.

PERFORMANCE CHARACTERISTICS

1. Immunoreactivity of CONFIRM anti-PR (1E2) was determined by a study that showed appropriate staining of PR antigen. The 90 normal tissues examined included: adrenal, bone marrow, breast, cerebellum, cerebrum, cervix, colon, endometrium, esophagus, heart, kidney, liver, lung, mesothelium, ovary, pancreas, parathyroid, peripheral nerve, pituitary, prostate, salivary gland, skeletal muscle, skin, small intestine, spleen, stomach, testis, thyroid, tonsil, and thymus. Staining was nuclear, with one case of ovary showing unexpected negative staining.

   Positive nuclear staining included the lobular and ductal cells of the breast, the glandular epithelium and fibromuscular cells of the cervix, the glandular epithelium, stromal tissues, and smooth muscle cells of the endometrium, and secretory cells of the adenohypophysis of all three cases of the pituitary tissues. Positive staining of thyroid tissue was observed, but this has been identified previously.[12]

   Ventana also tested a total of 54 formalin-fixed, paraffin-embedded neoplastic tissues with CONFIRM anti-PR (1E2), using the same protocols and pretreatment procedures as those used for the normal tissue testing. The tissues examined included neoplastic tissue from the following tissues: lung, prostate, colon, lymphoma, stomach, cervix, ovary, brain, pancreas, testis, thyroid, rectum, breast, spleen, esophagus, liver and kidney. For cervix, a single adenocarcinoma stained positive. For ovary, a single papillary adenocarcinoma contained positive staining nuclei. For thyroid, one medulary and one papillary carcinoma contained positive staining nuclei. A single islet cell carcinoma of the pancreas was positive. A single malignant mesenchymoma of the rectum was positive. A single adenocarcinoma of the prostate was positive. Both a leiomyoma and an adenocarcinoma of the uterus were positive for PR.

   Sensitivity is dependent upon the preservation of the antigen. Any improper tissue handling during fixation, sectioning, embedding or storage which alters antigenicity weakens PR detection by CONFIRM anti-PR (1E2) and may generate false negative results.
2. Six individual tissues cases were stained as part of the repeatability testing. Of the six tissues, two had PR high expression, two PR low expression, and two PR negative based on a cutoff of $\leq$1% tumor cells staining for negative, 1-10 % for low and >10% for high expression.

   For intra-day repeatability (intra-run) testing, 9 slides from each case were stained with CONFIRM anti-PR (1E2) antibody, and one slide from each case was stained with CONFIRM Negative Control Rabbit Ig antibody on a BenchMark XT instrument. The same testing configuration was also performed on a BenchMark ULTRA instrument.

   For day to day repeatability (inter-run) testing, four slides from each case were stained with the CONFIRM anti-PR (1E2) antibody, and one slide from each case was stained with CONFIRM Negative Control Rabbit Ig antibody in five separate non-consecutive runs conducted over a 20 day period on the same BenchMark XT instrument. The same testing configuration was also performed on a BenchMark ULTRA instrument.

   For intra-platform BenchMark XT instrument testing, 4 slides from six cases were stained with CONFIRM anti-PR (1E2) antibody across three separate BenchMark XT instruments. A single slide from each case was stained with CONFIRM Negative Control Rabbit Ig antibody.

   For intra-platform BenchMark ULTRA instrument testing, 4 slides from six cases were stained with CONFIRM anti-PR (1E2) antibody across three separate BenchMark ULTRA instruments. A single slide from each case was stained with CONFIRM Negative Control Rabbit Ig antibody.

   All reproducibility testing met the acceptance criteria for passing.
3. Comparison of CONFIRM anti-PR (1E2) to FLEX anti-PR (PgR 636).

   A randomized, multi-site, multi-reader study was conducted to compare the staining performance of the CONFIRM anti-PR (1E2) antibody on the BenchMark ULTRA instrument and on the BenchMark XT instrument to that of the Dako FLEX Monoclonal Mouse Anti-Human Progesterone Receptor Clone PgR 636 Ready-To-Use (FLEX anti-PR (PgR 636)) on the Dako Autostainer Plus. On the BenchMark instruments, endogenous biotin was blocked using the VENTANA Endogenous Biotin Blocking Kit. The antibody was detected using *i*VIEW DAB Detection Kit. On the Dako platform, the antibody was detected using EnVision Flex, High pH detection. Approximately 120 negative and 216 positive cases of breast cancer, representing the clinical range of the assay, were randomly assigned to three study sites such that each site received an equal number of cases and each site received cases representing each clinical assessment category. Each site stained its allotted cases with the CONFIRM anti-PR (1E2) antibody on a BenchMark ULTRA instrument, CONFIRM anti-PR (1E2) antibody on a BenchMark XT instrument, and with the FLEX anti-PR (PgR 636) on a Dako Autostainer Plus. The stained slides were evaluated by pathologists who determined the percentage of stained tumor cells. A case was considered PR positive if there was staining of the nucleus in at least $\geq$1% of invasive tumor cells.[19]

Table 3. CONFIRM anti-PR (1E2) on the BenchMark ULTRA Instrument Compared to FLEX anti-PR (PgR 636) on the Dako Autostainer Plus.

CONFIRM anti-PR (1E2)	FLEX anti-PR (PgR 636)		
	Positive	Negative	Total
Positive:	200	7	207
Negative:	9	104	113
Total:	209	111	320
	n/N	% (95% CI)	
Positive percent agreement	200/209	95.7 (92.0-97.7)	
Negative percent agreement	104/111	93.7 (87.6-96.9)	
Overall percent agreement	304/320	95.0 (92.0-96.9)	

Table 4. CONFIRM anti-PR (1E2) on the BenchMark XT Instrument Compared to FLEX anti-PR (PgR 636) on the Dako Autostainer Plus.

CONFIRM anti-PR (1E2)	FLEX anti-PR (PgR 636)		
	Positive	Negative	Total
Positive:	186	9	195
Negative:	18	100	118
Total:	204	109	313
	n/N	% (95% CI)	
Positive percent agreement	186/204	91.2 (86.5-94.3)	
Negative percent agreement	100/109	91.7 (85.0-95.6)	
Overall percent agreement	286/313	91.4 (87.7-94.0)	

Table 5. CONFIRM anti-PR (1E2) on the BenchMark ULTRA Instrument Compared to CONFIRM anti-PR (1E2) on the BenchMark XT Instrument.

BenchMark ULTRA Instrument	BenchMark XT Instrument		
	Positive	Negative	Total
Positive:	184	12	196
Negative:	6	105	111
Total:	190	117	307
	n/N	% (95% CI)	
Positive percent agreement	184/190	96.8 (93.3-98.5)	
Negative percent agreement	105/117	89.7 (82.9-94.0)	
Overall percent agreement	289/307	94.4 (90.9-96.3)	

For CONFIRM anti-PR (1E2) staining on the BenchMark ULTRA and BenchMark XT instruments compared to FLEX anti-PR (PgR 636) on the Dako Autostainer Plus, the positive, negative, and overall agreement rates (pooled across all sites) were all greater than 90%. For CONFIRM anti-PR staining on the BenchMark ULTRA instrument compared to the BenchMark XT instrument, the positive, negative, and overall agreement rates were all greater than 89%.

The morphology acceptability rates for all slides stained in this study were 99.7% (95% C.I. 98.3%-99.9%) on the BenchMark ULTRA instrument and 96.1% (95% C.I. 93.5%-97.7%) on the BenchMark XT instrument. The background acceptability rates were 99.4% (95% C.I. 97.9%-99.8%) on the BenchMark ULTRA instrument and 95.2% (95% C.I. 92.4%-97.0%) on the BenchMark XT instrument.

4. Comparison of *i*VIEW DAB Detection Kit and *ultra*View Universal DAB Detection Kit using CONFIRM anti-PR (1E2).

CONFIRM anti-Progesterone Receptor (PR) (1E2) Rabbit Monoclonal Primary Antibody was used to conduct detection comparison testing across two instruments (BenchMark XT and BenchMark ULTRA instrument) using *i*VIEW DAB Detection Kit and *ultra*View Universal DAB Detection Kit. One hundred and ninety nine tissue cases were used as part of the testing. Of the cases, approximately half are positive and half negative as a function of percentage of tumor cells stained. The stained slides were evaluated by pathologists who determined the percentage of stained tumor cells. A case was considered PR positive if there was staining of the nucleus in at least 1% of tumor cells.

The morphology and background acceptability rates were 100% for both detection kits and instruments except for *ultra*View Universal DAB Detection Kit on the BenchMark ULTRA instrument, which had a background acceptability rate of 99.5. Direct comparisons for positive and negative clinical assessment between detection kits for each platform are presented in Table 6 for the BenchMark ULTRA instrument and Table 7 for the BenchMark XT instrument.

Table 6. Clinical assessment for *ultra*View Universal DAB Detection Kit vs. *i*VIEW DAB Detection Kit with the BenchMark ULTRA Instrument.

*ultra*View Universal DAB Detection Kit	*i*VIEW DAB Detection Kit		
	Positive	Negative	Total
Positive	94	11	105
Negative	2	86	88
Total	96	97	193
	n/N	% (95% CI)	
Positive percent agreement	94/96	97.9 (92.7-99.4)	
Negative percent agreement	86/97	88.7 (80.8-93.5)	
Overall percent agreement	180/193	93.3 (88.8-96.0)	

Table 7. Clinical assessment for *ultra*View Universal DAB Detection Kit vs. *i*VIEW DAB Detection Kit with the BenchMark XT Instrument.

*ultra*View Universal DAB Detection Kit	*i*VIEW DAB Detection Kit		
	Positive	Negative	Total
Positive	91	14	105
Negative	2	86	88
Total	93	100	193
	n/N	% (95% CI)	
Positive percent agreement	91/93	97.8 (92.5-99.4)	
Negative percent agreement	86/100	86.0 (77.9-91.5)	
Overall percent agreement	177/193	91.7 (87.0-94.8)	

Agreement of clinical assessment between detection kits for both instruments was above 90% at 93.3% (n=193) and 91.7% (n=193) for BenchMark ULTRA and BenchMark XT instruments respectively. The *ultra*View Universal DAB Detection Kit compared to *i*VIEW DAB Detection Kit had staining score agreement rates of 90.2% (n=193) and 85.5% (n=193).

TROUBLESHOOTING

1. If the positive control exhibits weaker staining than expected, other positive controls run concurrently should be checked to determine if it is due to the primary antibody or one of the common secondary reagents.
2. If the positive control is negative, it should be checked to ensure that the slide has the proper barcode label. If the slide is labeled properly, other positive controls run concurrently should be checked to determine if it is due to the primary antibody or one of the common secondary reagents. Tissues may have been improperly collected, fixed or deparaffinized. The proper procedure should be followed for collection, storage and fixation.

3. If excessive background staining occurs, high levels of endogenous biotin may be present. A biotin blocking step should be included.
4. If all of the paraffin has not been removed, the deparaffinization procedure should be repeated.
5. If specific antibody staining is too intense, the run should be repeated with the primary antibody incubation time shortened by 4 minute intervals until the desired stain intensity is achieved.
6. If tissue sections wash off the slide, slides should be checked to ensure that they are positively charged.
7. For corrective action, refer to the Step By Step Procedure section of the automated slide stainer Operator's Manual or contact your local support representative.

REFERENCES

1. Bevitt DJ, Milton ID, Piggot N, Henry L, Carter MJ, Toms GL, Lennard TW, Westley B, Angus B, Horne CH. New Monoclonal antibodies to oestrogen and progesterone receptors effective for paraffin section immunohistochemistry. J Pathol. 1997;183(2):228-232.
2. Dickson RB, Lippman ME. Genes, oncogenes, and hormones: advances in cellular and molecular biology of breast cancer. Kluwer Academic Publishers, Boston, 1992: 285-286.
3. Rocha R, Nunes C, et al. Rabbit monoclonal antibodies show higher sensitivity than mouse monoclonals for estrogen and progesterone receptor evaluation in breast cancer by immunohistochemistry. Pathol Res Pract. 2008;204(9):655-62.
4. Cano G, Milanezi F, et al. Estimation of hormone receptor status in fine-needle aspirates and paraffin-embedded sections from breast cancer using the novel rabbit monoclonal antibodies SP1 and SP2. Diagn Cytopathol. 2003;29(4):207-11.
5. Rhodes A, Sarson J, et al. The reliability of rabbit monoclonal antibodies in the immunohistochemical assessment of estrogen receptors, progesterone receptors, and HER2 in human breast carcinomas. Am J Clin Pathol. 2010;134(4):621-32.
6. O'Connor, SM, Beriwal S, et al. Concordance between semiquantitative immunohistochemical assay and oncotype DX RT-PCR assay for estrogen and progesterone receptors. Appl Immunohistochem Mol Morphol. 2010;18(3):268-72.
7. Wei LL, Miner R. Evidence for the existence of a third progesterone receptor protein in human breast cancer cell line T47D. Cancer Res. 1994;54(2):340-3.
8. Doglioni C, Gambacorta M, Zamboni G, Coggi G, Viale G. Immunocytochemical localization of progesterone receptors in endocrine cells of the human pancreas. Am J Pathol. 1990;137(5):999-1005.
9. Greene GL, Press MF. Immunochemical evaluation of estrogen receptor and progesterone receptor in breast cancer. In: Ceriani RL, editor. Immunological Approaches to the Diagnosis and Therapy of Breast Cancer. New York: Plenum Press, 1987: 119-135.
10. Press MF, Greene GL. Localization of progesterone receptor with monoclonal antibodies to the human progestin receptor. Endocrinology. 1988;122(3):1165-1175.
11. Winborn WB, Sheridan PJ, McGill HC Jr. Localization of progestin receptors in the islets of Langerhans. Pancreas. 1987;2(3):289-94.
12. Money SR, Muss W, Thelmo WL, Boeckl O, Pimpl W, Kaindl H, Sungler P, Kirwin J, Waclawicek H, Jaffe BM, et al. Immunocytochemical localization of estrogen and progesterone receptors in human thyroid. Surgery. 1989;106(6):975-8.
13. Roche PC. Immunohistochemical stains for breast cancer. Mayo Clin Proc. 1994;69(1):57-58.
14. PDQ. Treatment. Health Professionals. Breast Cancer. Downloaded from http://icisun.nci.nih…t_cancer_Physician.html on 4/10/1997.
15. Horwitz KB. How do breast cancers become hormone resistant? J Steroid Biochem Mol Biol. 1994;49(4-6):295-302.
16. Van Agthoven T, Timmermans M, Dorssers LC, Henzen-Logmans SC. Expression of estrogen, progesterone and epidermal growth factor receptors in primary and metastatic breast cancer. Int J Cancer. 1995;63(6):790-793.
17. Horwitz KB, McGuire WL. Predicting response to endocrine therapy in human breast cancer: a hypothesis. Science. 1975;189(4204):726-727.
18. American Society of Clinical Oncology. Clinical practice guidelines for the use of tumor markers in breast and colorectal cancer. J Clin Oncol. 1996;14(10):2843-2877.
19. Hammond ME, Hayes DF, et al. American Society of Clinical Oncology/College of American Pathologists guideline recommendations for immunohistochemical testing of estrogen and progesterone receptors in breast cancer. Arch Pathol Lab Med. 2010;134(6):907-22.
20. ODE Guidance documents, Center for Devices and Radiological Health, updated 7/29/1996. Draft: Premarketing approval review criteria for premarket approval of estrogen (ER) or progesterone (PGR) receptors in vitro diagnostic devices using steroid hormone binding (SBA) with dextran coated-charcoal (DCC) separation, histochemical receptor binding assays, or solid phase enzyme immunoassay (EIA) methodologies.
21. Carson F, Hladik C. Histotechnology: A Self Instructional Text, 3rd edition. Hong Kong: American Society for Clinical Pathology Press; 2009.
22. College of American Pathologists Laboratory Accreditation Program, Anatomic Pathology Checklist, 2010.
23. CLSI. Quality Assurance for Immunocytochemistry: Approved Guideline. CLSI document MM4-A- (ISBN 1-56238-396-5). CLSI, 940 West Valley Road, Suite 1400, Wayne, PA 19087-1898 USA, 1999.
24. Nadji M, Morales AR. Immunoperoxidase: part 1. The technique and its pitfalls. Lab Med. 1983;14:767.
25. Herman GE, Elfont EA. The taming of immunohistochemistry: the new era of quality control. Biotech Histochem. 1991;66(4):194-199.
26. Omata M, Liew CT, Ashcavai M, Peters RL. Nonimmunologic binding of horseradish peroxidase to hepatitis B surface antigen. A possible source of error in immunohistochemistry. Am J Clin Pathol. 1980;73(5):626-32.
27. Liu S, Chia SK, et al. Progesterone receptor is a significant factor associated with clinical outcomes and effect of adjuvant tamoxifen therapy in breast cancer patients. Breast Cancer Res Treat. 2010;119(1):53-61.
28. Qiu J, Kulkarni S, et al. Effect of delayed formalin fixation on estrogen and progesterone receptors in breast cancer: a study of three different clones. Am J Clin Pathol. 2010;134(5):813-9.

INTELLECTUAL PROPERTY

CONFIRM, BENCHMARK, *ultra*View, VENTANA, and the VENTANA logo are trademarks of Roche.

All other trademarks are the property of their respective owners.

Ventana Medical Systems, Inc. grants to the Purchaser a single use only license under U.S. Pat. Nos. 6045759, 6945128, and 7378058, and any foreign counterparts.

© 2011 Ventana Medical Systems, Inc.

CONTACT INFORMATION

Ventana Medical Systems, Inc.
1910 E. Innovation Park Drive
Tucson, Arizona 85755
USA
+1 520 887 2155
+1 800 227 2155 (USA)

www.ventana.com

EC REP

Roche Diagnostics GmbH
Sandhofer Strasse 116
D-68305 Mannheim
Germany

CE
