# Supplementary material for: High Progesterone Receptor Expression in Prostate Cancer Is Associated with Clinical Failure
Source: PLoS One. 2015 Feb 27;10(2):e0116691. doi: 10.1371/journal.pone.0116691 (PMC4344236; doi:10.1371/journal.pone.0116691)
Supplement: S3 Text — (DOCX) [file pone.0116691.s006.docx]

**Ki67 immunostaining**

In retrospect of the initial PGR analyses the prostate specimens were stained for Ki67 to evaluate if any correlation between Ki67 and PGR expression and other clinopathological variables could be detected.

**Quantification of Ki67 immunostaining.**

The following antibody from Ventana Medical (Tucson, Arizona, USA) was applied to assess the proliferative activity of normal and neoplastic tissue: CONFIRM Ki-67 (30-9) rabbit monoclonal primary antibody directed against the C-terminal portion of the Ki67 antigen. The applied antibody is produced for routine diagnostic IHC and has received FDA approval (510k) for IVD (*in vitro* diagnostic) use. Ki67 positive staining was identified by the presence of brown nuclear (DAB) staining in tumor cells. KI-index was quantified using The ARIOL imaging system (Applied Imaging Corp., San Jose, CA, USA) and the percentage of stained tumor cells of the total number of at least 200 tumor cells were counted for each core and scored using the following system: 0 = 0 %, 1 = 1 – 2.5 %, 2 = 2.6 – 4, 3 ≥ 5%. The scoring values were then dichotomised as low or high expression. A high expression was defined as scoring values above 4^th^ quartile (≥ 1). Details regarding TMA, IHC and scoring system is listed in the methods section of the main manuscript.

**Results**

**Ki67 expression and correlation with PGR and clinicopathological variables**

Positive Ki67 staining was clearly detected in the nucleus of TE in 321 (60 %) of the 535 patients and 184 (57.3 %) of these cases had a Ki67 expression ≥ 1. A weak, but significant correlation was detected between Ki67 and PGR expression (r = 0.12, p = 0.012). No correlation between Ki67 and other clinopathological variables was detected.

**Univariate analyses**

Ki67 expression was significantly associated with CFFS (p = 0.012). Ten year CFFS were 95.3 % vs. 86.0 % respectively for patients with low expression vs. those with high. When merging PGR and Ki67 expression, patients with high (high/low, low/high, and high/high) expression had significantly reduced CFFS (p = 0.004) compared to those with low (low/low) expression. Ten year CFFS were 96.9 % vs. 88.4 % respectively for patients with low (low/low) expression vs. those with high (low/high, high/low and high/high).

**Multivariate analyses**

In the multivariate analysis, a high (high/low, low/high, and high/high) combined expression of Ki67 and PGR was an independent predictor for CF (HR: 3.9, 95 % CI: 1.3 – 12.0, p = 0.015).
